# Comparative Microstructural Evaluation of Wood in Three Dominant *Ziziphus* Species of Desert Ecosystem (Cholistan), Pakistan

**DOI:** 10.1155/2024/3323920

**Published:** 2024-09-25

**Authors:** Muhammad Bilal, Zaheer-ud-din Khan, Sohaib Muhammad, Uzma Hanif, Khalid Hussain, Muhammad Tayyab, Andleeb Anwar Sardar, Hassan Nawaz, Muhammad Jawad Tariq Khan, Mahrukh, Aneela Rasool, Summiya Faisal, Muhammad Zahid

**Affiliations:** ^1^ Dendrochronology Lab Department of Botany Government College University, Lahore 54000, Pakistan; ^2^ Pakistan Forest Institute, Peshawar, Pakistan

## Abstract

The present microstructural evaluation was carried out on the woods of three ethnobotanically important local fruit trees, namely, *Ziziphus mauritiana* Lam., Z. *spina-christi* (L.) Willd., and *Z. nummularia* (Burm.f.) Wight and Arn., of family Rhamnaceae from Cholistan Desert of Pakistan. Wood samples were sectioned with sliding sledge microtome to make permanent slides for observing different anatomical parameters under the light microscope. All selected species were observed to have diffuse-porous wood with indistinct growth rings. The vessels were rounded in outline in all the species studied and found mostly solitary or in radial multiples of 2 in *Ziziphus mauritiana* and *Z. nummularia*, while in radial multiples of 2 to 5 in *Z. spina-christi*. The intervessel pits were scalariform to opposite. The rays were uniseriate in *Ziziphus mauritiana*, while mostly were biseriate in *Ziziphus spina-christi*. Simple perforation plates and diffuse, confluent, and vasicentric types of axial parenchyma were present in all the selected species. The fibers were thin-walled and nonseptate. One-way ANOVA followed by the Tukey test was conducted to observe different anatomical variations within selected species. Principal component analysis revealed correlations among studied anatomical parameters. The number of rays per mm was comparatively larger in *Ziziphus nummularia,* showing its greater susceptibility to wood-deteriorating agents than in other selected species. The Runkel ratio indicated the selected species suitable for making paper.

## 1. Introduction

The microstructural evaluation of wood provides inspirational and fascinating data that are applicable to various botanical disciplines, such as systematics, ecology, paleobotany, and physiology [1, 2]. Wood anatomy is the mirror image of the climatic fluctuations in that geographical region [3–5]. The wood anatomical characteristics vary according to the climatic nature of the region [6–10]. Because structural changes can be studied under different environmental conditions, comparative wood anatomy is useful in evolutionary studies for ecological strategies [11–14]. The plants develop various anatomical adaptations in different environmental conditions to accomplish certain functions [15–17].

The Cholistan Desert of Punjab, having an area of 26000 km^2^, is located south of Bahawalpur in Punjab and extends across the Thar and Nara Deserts of Sindh having vegetation of xerophytic species [18]. *Ziziphus* spp. (Genus: *Ziziphus*, Family: Rhamnaceae, Order: Rosales, Class: Magnoliopsida, Phylum: Tracheophyta) are the indigenous and dominant woody trees of the Cholistan Desert. Locally, the wood of *Ziziphus* spp. is commonly used in village dwellings and furniture making, while their bark, leaves, and fruits are used in folk medicines. The honeybees collect nectar from their flowers, while the birds feed on their fruits. By considering its importance in the local industry, the present research was conducted to evaluate their wood anatomical properties to make its usage at commercial level. Wood anatomical characteristics of the genus *Ziziphus* species have been studied in various regions of the world [19–22]. The microstructural evaluation has not been conducted so far on the wood of *Ziziphus* spp. from Cholistan Desert of Pakistan. So, the present research on the microstructural evaluation of three species of the genus *Ziziphus*, i.e., *Z. spina-christi*, *Z. mauritiana*, and *Z. nummularia* of the Cholistan Desert of Pakistan was conducted to record their comparative anatomical characteristics in that geographical area.

There is also a need to find some alternate proper source of wood for the local industry that may reduce the pressure on Teak and Sheesham woods, profusely used in the local furniture industry, being the best ideal wood. Therefore, the present comparative wood anatomical studies are conducted to ensure the usage of these woods at the commercial level. Through anatomical study, we can determine the age, growth rate, growth developmental pattern in trees, and the comparative nature of geographical areas of wood collection sites [23].

This research work highlights the anatomical properties of targeted woody trees of the study area. Wood quality refers to the cumulative effect of wood characteristics on the anatomical cellular, and chemical features of the wood within and among trees. Thus, anatomical studies on wood are conducted for tree improvement programs to enhance wood quality [24]. Furthermore, the wood quality has a significant impact on wood processing, end-product quality, and marketing [25]; hence, it is helpful for foresters and the whole timber industry, giving a broad range of options for the construction sector and the furniture industry. The present investigation aimed to study the wood anatomical characteristics of three *Ziziphus* spp. of the Cholistan Desert to evaluate their better usage at the commercial level for paper and pulp manufacturing and the furniture industry. Moreover, this research work will be helpful in the wood identification of studied plants.

## 2. Materials and Methods

### 2.1. Sample Collection

**Figure 1:**
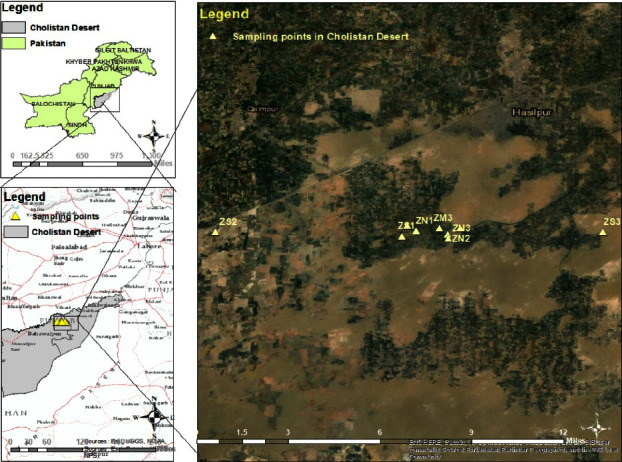
Map of sampling points in the Cholistan Desert, Pakistan.

Three wood samples of each species of genus *Ziziphus* Mill., i.e., *Z. spina-christi*, *Z. mauritiana*, and *Z. nummularia*, were collected from different locations in the Cholistan Desert, Pakistan, as illustrated in Table 1 (Figure 1). Trees were selected from distant locations away from urban areas to minimize the effects of harmful air pollution and allow for accurate wood anatomical assessment in that geographical area. The authentic and average samples (wood discs) were taken from the breast height of the tree.

*Ziziphus mauritiana* is a large tree or shrub with drooping, spreading branches. The leaves have a tomentose underside and a glossy, larger (2–9 cm) surface. It has bigger fruits (3.5–1.5 cm). *Ziziphus nummularia* is a dense shrub with tomentose, divaricating branches. The undersides of the leaves are velvety tomentose, thickly pubescent, and smaller (1–2 cm). It has tiny, globose fruits (5–10 mm) that, when ripe, change from reddish-brown to black. The medium-sized tree *Ziziphus spina-christi* has spreading, greyish-white branches. The flowers are greenish-yellow, and the leaves are glabrous or pubescent, measuring 2–6 cm. The globose fruits resemble *Z. nummularia'*s in size but are slightly elongated [26].

### 2.2. Section Softening, Cutting, and Staining

**Figure 2:**
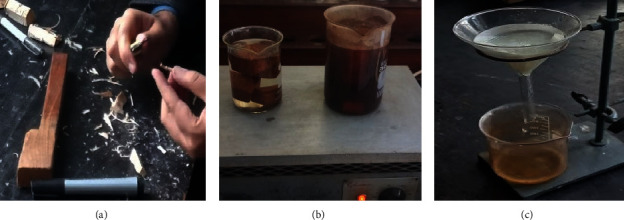
Preparation of samples for maceration (a). Boiling of samples by using hot plate (b). Washing of macerated material (c).

The standard procedure for wood softening and sectioning was used in the present study at PFI (Pakistan Forest Institute), Peshawar, KP. Prior to sectioning, wood samples were made softened by boiling them in water for 21 to 22 hours (Figure 2(b)). Then, sectioning was accomplished by cutting wood samples into three planes, i.e., cross section, radial longitudinal section, and tangential longitudinal section with sliding sledge microtome. After staining with safranin, aniline blue, or fast green, permanent slides were prepared by using Canada balsam [27, 28].

### 2.3. Maceration of Wood

From the radial side, small pieces of wood (matchstick size) were taken (Figure 2(a)) and macerated them in potassium chlorate solution and 20 per cent nitric acid for 5–6 hours using the water bath for boiling until they become soft and white [22]. The macerated material was then stained in aniline blue or safranin after thoroughly washing with water (Figure 2(c)) [28].

### 2.4. Microscopy

The light microscope was used for the examination of prepared microscopic slides to record different wood parameters, i.e., fiber length, fiber width, fiber wall thickness, lumen diameter, ray height and diameter, number of cells along ray width and height, vessel length, and diameter at different magnifications. 100 random fibers were selected for measuring their various parameters like fiber length, width, wall thickness, and lumen diameter. 50 vessel and rays were randomly selected for measuring their length and diameter, no. of cells along ray height, and no. of cells along ray diameter. For vessel frequency and number of rays per mm, 10 fields were selected on cross section and tangential longitudinal section. Dimensions of various anatomical parameters were taken with the help of WinCell system software. The anatomical descriptions and terminologies were used by following IAWA (International Association of Wood Anatomists) list of microscopic features for hardwood identification [29].

## 3. Results

The detailed anatomical characteristics of the wood of targeted three species of the genus *Ziziphus* are presented below.

### 3.1. *Ziziphus mauritiana* Lam

**Anatomical features**—Diffuse porous, indistinct growth rings.

**Figure 3:**
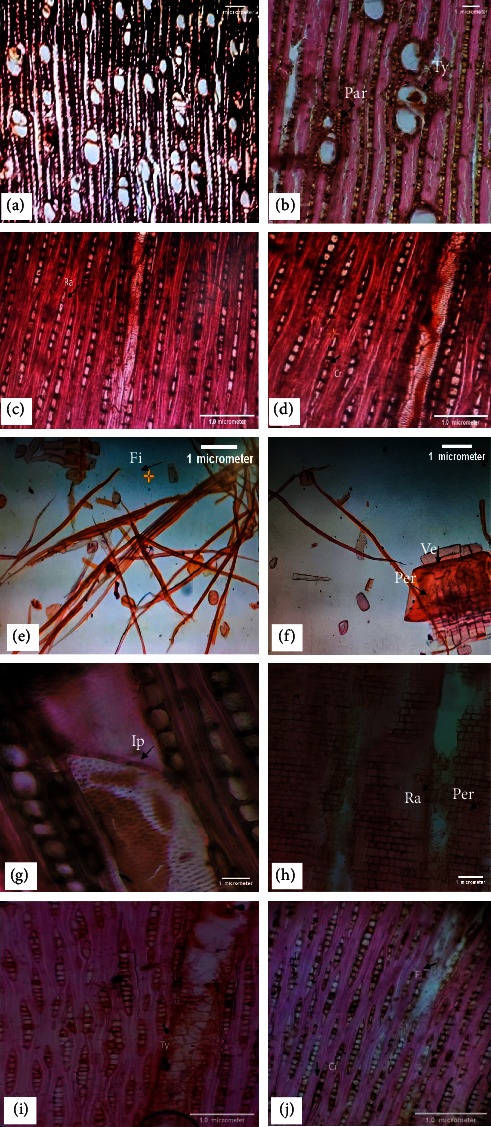
(a-j). *Z. mauritiana*: cross section (4x)—wood is diffused porous having mostly solitary vessels or in radial multiple of two or three, tyloses (Ty) in vessels (a); cross section (10x)—vasicentric type of parenchyma (par) with narrow sheath (b); tangential longitudinal section (10x)—uniseriate rays (Ra) (c); tangential longitudinal section (10x)—crystals (cr) in rays (d); macerated material (4x)—fibers (Fi) (e); macerated material (10x)—long-tailed vessel (Ve) with perforation plate (per) simple (f); tangential longitudinal section (40x)—scalariform to opposite intervessel pits (Ip) (g); radial longitudinal section (10x)—homogenous with square ray (Ra) cells (h), perforation plate (per) simple; tangential longitudinal section (10x)—tyloses (ty) present in vessels (i); fibers (fi) without septation, crystals (cr) in rays (j).

**Vessels**—The mean tangential diameter of the vessel is 139.23 *µ*m, mostly solitary or in a radial multiple of two or three, with a rounded outline having tyloses (Figure 3(a) and 3(i)), simple perforation plates (Figure 3(h) and 3(f)), and scalariform to opposite intervessel pits (Figure 3(g)), having a 6.01 mm^2^ mean vessel frequency.

**Fibers—**Nonseptate (Figure 3(j)) and thin-walled, with a mean wall thickness of 4.22 *µ*m, 930.8 *µ*m mean fiber length, and 14.52 *µ*m mean fiber width, while the mean lumen diameter is 10.33 *µ*m (Figure 3(e)).

**Parenchyma**—Axial parenchyma is of the vasicentric type, with strands of 8–13 cells and 1–3 cells wide (Figure 3(b)).

**Rays**—Uniseriate with mean ray height is 283.01 *µ*m, 27.44 *µ*m mean diameter, 6.66 numbers of cells along the ray height, while 1 cell is along the ray width and 17.86 mm ray frequency (Figure 3(c)). Homogenous, with all ray cells square in outline (Figure 3(h)) and containing crystals (Figure 3(d)).

### 3.2. *Ziziphus spina-christi* (L.) Willd

**Anatomical features—**Diffuse porous, indistinct growth rings.

**Figure 4:**
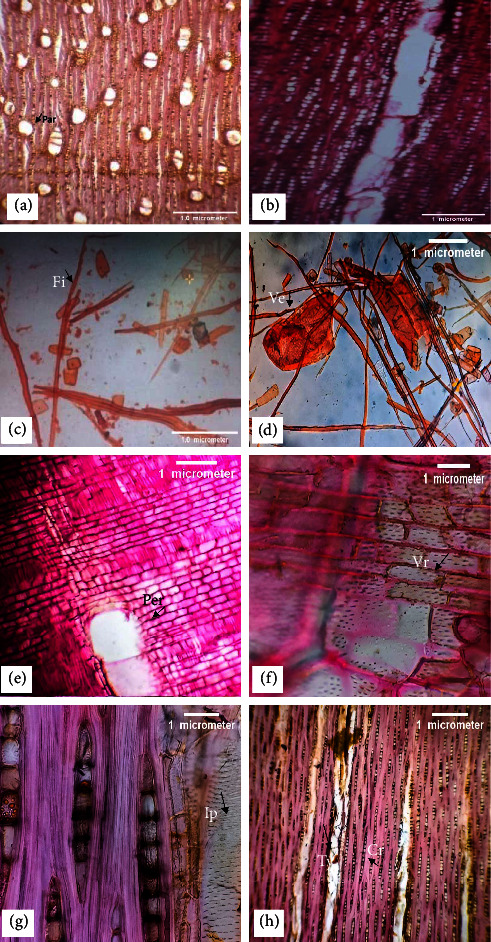
(a-h). *Z. spina-christi*: cross section (4x)—diffused porous having solitary vessels or in radial multiples of five with vasicentric type of parenchyma (par) (a); tangential longitudinal section (10x)—uniseriate and biseriate both types of rays and fibers without septation (b); macerated material (10x)—libriform type of fibers (c); macerated material (10x)—long-tailed vessels (Ve) with large intervessel pits (d); radial longitudinal section (10x)—perforation plate (Per) simple, heterogenous with square and upright ray cells (e); radial longitudinal section (40x)—vessel-ray (Vr) pitting with distinct border (f); tangential longitudinal section (40x)—scalariform to opposite type of intervessel pits (Ip) (g); tangential longitudinal section (4x)—tyloses (Ty) in vessels and crystals (Cr) in rays (h).

**Vessels—**The mean tangential diameter of the vessel is 159.58 *µ*m, with intervessel pits alternate (Figure 4(g), and the mean vessel frequency is 3.45 mm^2^. Solitary or in a radial multiple of two to five, simple perforation plates, and rounded in outline (Figure 4(a), 4(d)).

**Fibers—**Nonseptate (Figure 4(b)) and thin-walled, with a mean fiber length of 1171.53 *µ*m and a mean fiber width of 19.68 *µ*m. The mean fiber wall thickness is 4.33 *µ*m, and the mean lumen diameter is 10.16 *µ*m (Figure 4(c)).

**Parenchyma—**Axial parenchyma is of the vasicentric type, with strands of 5–9 cells and 1–3 cells wide (Figure 4(a)).

**Rays—**Uniseriate to biseriate (Figure 4(b)) with a 250.82 *µ*m mean ray height while the mean diameter is 12.47 *µ*m. 7.53 numbers of cells along the ray height, 1–3 cells along the ray width, and a ray frequency of 20.60 mm. Heterogenous type of ray cells with square and upright outline (Figure 4(e)). Vessel-ray pitting with distinct border (Figure 4(f)) and crystals present in rays (Figure 4(h)).

### 3.3. *Z. nummularia* (Burm.f.) Wight and Arn

**Anatomical features**—Diffuse porous, growth rings indistinct.

**Figure 5:**
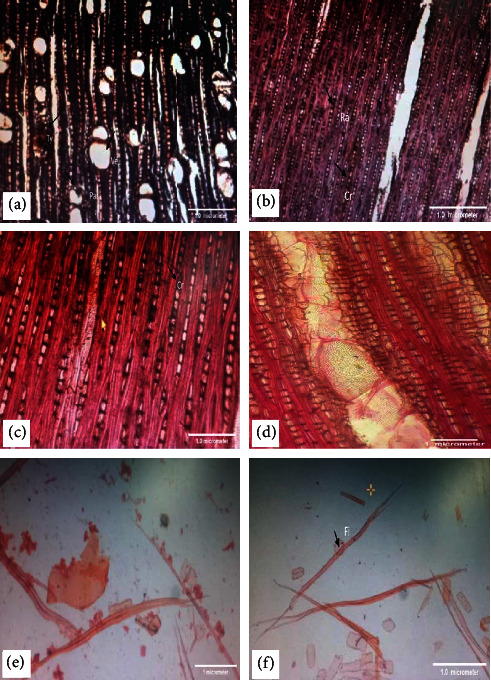
(a-f). *Z. nummularia*: cross section (10x)—diffused porous with solitary vessels (Ve) or multiples of two or three, rounded outline of vessels, tyloses (Ty) in vessels, vasicentric, diffuse, confluent type of parenchyma (Par) (a); tangential longitudinal section (4x)—uniseriate type of rays (ra) with crystal (Cr) (b); tangential longitudinal section (10x)—crystals (Cr) in rays (c); tangential longitudinal section (40x)—opposite type of intervessel pits (Ip) (d); macerated material (10x)—fibers and vessel fragment (e); macerated material (4x)—fibers (Fi) (f).

**Vessels**—One or in a radial multiple of two having tyloses in some vessels (Figure 5(a)), simple perforation plates, rounded in outline, and the mean tangential diameter of the vessel is 144.29 *µ*m. Intervessel pits are opposite to alternate (Figure 5(d)). The vessel frequency is 9.53 mm^2^.

**Fiber—**Fibers (Figure 5(e), 5(f)) are thin-walled, with a mean fiber wall thickness of 4.25 *µ*m, a mean length of 958.36 *µ*m, a mean width of 14.01 *µ*m, a lumen diameter of 9.53 *µ*m, and nonseptate (Figure 5(c)).

**Parenchyma—**Axial parenchyma is of diffuse, confluent, and vasicentric type, with strands of 5–17 cells and 1–3 cells wide (Figure 5(a)).

**Rays—**Rays uniseriate (Figure 5(b)) with a mean ray height of 238.83 *µ*m and a mean ray diameter of 24.88 *µ*m. The 9.06 numbers of cells along the ray height and the ray width are 1, and the ray frequency is 34.33 mm.

### 3.4. Comparative Analysis of Anatomical Features

**Figure 6:**
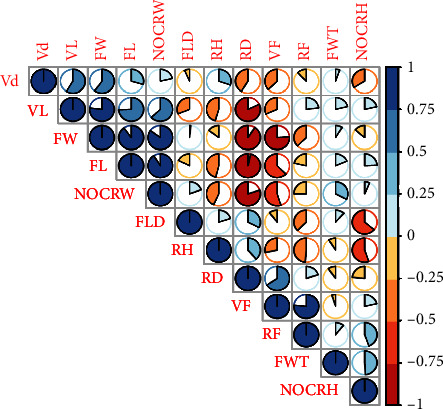
Correlation matrix among different anatomical parameters of targeted species. Vd = vessel diameter, VL = vessel length, FW = fiber width, FL = fiber length, NOCRW= number of cells along ray width, FLD = fiber lumen diameter, RH = ray height, RD = ray diameter, VF = vessel frequency, RF = ray frequency, FWT = fiber wall thickness, NOCRH = number of cells along ray height.

**Figure 7:**
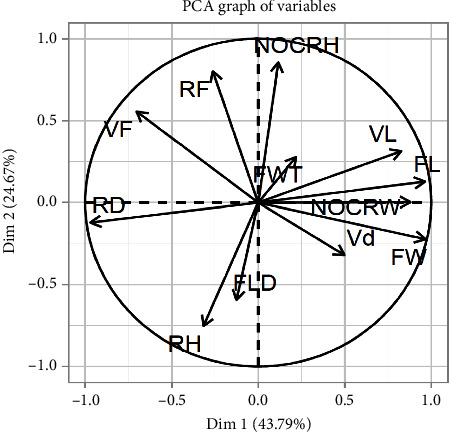
Principal component analysis (PCA) of anatomical features of three *Ziziphus* species. Vd = vessel diameter, VL = vessel length, FW = fiber width, FL = fiber length, NOCRW = number of cells along ray width, FLD = fiber lumen diameter, RH = ray height, RD = ray diameter, VF = vessel frequency, RF = ray frequency, FWT = fiber wall thickness, NOCRH = number of cells along ray height.

One-way ANOVA, followed by the Tukey test (Tables 2, 3, and 4), revealed that some anatomical parameters have significant variations among species, i.e., *Z. spina-christi* has fiber length (*F* = 11.82, *P*=0.01), width (*F* = 54.95, *P*=less than 0.001), vessel length (*F* = 61.57, *P*=less than 0.001), and vessel frequency (*F* = 56.42, *P*=0.01) significantly different from the other two selected species. Vessel length (*F* = 61.57, *P*=less than 0.001) and frequency (*F* = 56.42, *P*=0.01) are significantly larger in *Z. mauritiana*. Ray frequency (*F* = 35.44, *P*=less than 0.001) and vessel frequency (*F* = 56.42, *P*=0.01) are significantly larger in *Z. nummularia.* Wider rays are present in *Z. mauritiana*. There is a strong correlation among different studied anatomical parameters: Pearson correlations describe the positive relationship of vessel length with other anatomical parameters, i.e., with vessel diameter (58% at *P*=0.048), fiber width (76% at *P*=0.008), and negative relationship with ray diameter (82% at *P*=0.003) as shown in Table 5. Some other parameters also have significant correlations, i.e., vessel diameter has positive correlation with fiber width (60% at *P*=0.04); vessel frequency is negatively correlated with fiber width (76% at *P*=0.009) and positively correlated with Ray frequency (76% at *P*=0.008) and ray diameter (65% at *P*=0.02), as described in Table 5 (Figure 6). The PCA biplot projects variables onto the first two main components to show the correlations between different variables. The direction and length of each arrow denote the variable's contribution to the principal components. Positive correlation exists between variables heading in the same direction (FL, VL, NOCRW, and FW), while negative correlation exists between variables pointing in opposite directions (RF and VF versus FL and VL). Variables like RF, VF, RD, and RH contribute negatively to Dim 1 but positively to Dim 2, as seen by this biplot, indicating that these components are influenced by distinct underlying factors. On the other hand, Dim 1 shows high positive contributions from variables such as FW, NOCRW, Vd, FL, and VL, showing their considerable influence on this dimension (Figure 7). Selected plants have a vulnerability and mesomorphy index greater than 1 and 200, respectively, indicating they are of tropical environment (Table 6). Arithmetic ratios of fiber parameters demonstrate the suitability of selected plant spp. for pulp and paper manufacturing, i.e., a Runkel ratio close to 1 (Table 7).

## 4. Discussion

The present study revealed a close relationship among all selected species of *Ziziphus*, i.e., diffuse porous with indistinct growth rings, having simple perforation plates. The findings of previous research from various parts of world [19–22] on the quantitative features including fiber length, width, wall thickness, lumen diameter, vessel diameter, vessel element length, ray height, and ray diameter of *Z. mauritiana*, *Z. nummularia,* and *Z. spins-christi* showed significant variations as compared with the findings of the present work. These variations in anatomical properties may be due to different climatic fluctuations of different geographical areas. Fibers are classified into three groups [29]: Short fibers with length less than 900, fibers of medium length between 900 and 1900 *µ*m, and fibers longer than 1900 *µ*m are regarded longer length fibers. All selected species have medium-length fibers, i.e., 1171.53 *µ*m, 958.36 *µ*m, and 930.8 *µ*m in *Z*. *spina-christi*, *Z*. *nummularia*, and *Z*. *mauritiana*, respectively. The fiber length and width of *Z. spina-christi* are significantly higher than that of the other two species. The ray height exceeding 1 mm and ray seriation >10 are generally regarded as larger rays [29]. In the present study, rays are mostly uniseriate or biseriate and ray height is not exceeding 1 mm. So, shorter rays are present in all selected plants. The Runkel ratio is directly influenced by cell wall thickness [30]. Thick-walled fibers have Runkel ratios higher than 1 producing bulky structures with higher porosity [31, 32]. The Runkel ratio lower than 1 describes fibers with more flexibility, quickly collapsing, forming a structure with good strength properties, i.e., suitable for fibrous networking and bonding. All selected species have a Runkel ratio lower than 1, so these are suitable for paper and pulp manufacturing (Table 7). The rigidity coefficient parameter is an indicator of bending resistance and is related to fiber flexibility. The rigidity coefficient ratio of *Z. nummularia* and *Z. spina-christi* is 30 and 21.33, respectively, while that of *Z. mauritiana* is 29.66 (Table 7). The flexibility coefficient being directly influenced by fiber wall thickness and diameter describes the fiber flexibility [33]. Fibers are classified into three categories: elastic fibers (flexibility ratio 50–75%), rigid fibers (flexibility ratio 30–50%), and high rigid fibers (<30% flexibility ratio) [34]. In the present study, *Z. mauritiana*, *Z. nummularia*, and *Z. spina-christi* have the values of 72, 69, and 50, respectively, showing higher fiber flexibility (Table 7). These calculated values of arithmetic ratios in fiber dimensions of selected spp. showing their suitability for paper and pulping manufacturing.

The anatomical properties help to assess wood's behavior and its suitability for different uses. The frequency and size of vessels have a correlation with the seasoning and preservation behavior of wood [35]. Results in the present work showed that all selected spp. are good in seasoning and preservation. Vulnerability and mesomorphy are two ecological indicators in wood anatomy [36–38]. Their lower values, i.e., <1 and 50, show xeric adaptability conditions, whereas high values, i.e., >1 and 800, show mesic adaptability. Due to their consistence with their tropical origin, the targeted wood samples showed high values of vulnerability and mesomorphy, i.e., >1 and 800.

The above studied anatomical characteristics, i.e., vessel morphology, shorter rays, medium fiber length, and width and thin-walled fibers, revealed their suitability for furniture making and other related purposes. The arithmetic ratios of selected three plant species, i.e., Runkel ratios lower than 1 and higher flexibility ratios, showed that these can be used for pulp and paper production.

## 5. Conclusions

The current study described the comprehensive detail of anatomical characteristics of selected species from Desert ecosystem (Cholistan), Pakistan. The anatomical features of fibers, vessels, rays, and parenchyma of selected three species were analyzed qualitatively and quantitatively. These distinguished anatomical parameters are useful guidelines for foresters in plant identification and their better usage in the furniture and wood product industry. Wood identification would provide a safeguard in avoiding the illegal lodging of these important tree species of desert. Arithmetic ratios in fiber dimensions of selected spp. showed their potentiality in the manufacturing of paper and pulp. The vessel dimensions of selected plant species, i.e., vessel length and diameter, indicate that these can behave better in the wood seasoning and preservation processes. Moreover, these findings will be helpful in tree improvement programs and conservation strategies of these important woody plants.

## Figures and Tables

**Table 1:** Edaphoclimatic characters of *Ziziphus* species of Cholistan Desert.

Location	Plant species	Lat	Long	Dbh (cm)	Soil type	Temp. range	Annual precip. (mm)
Cholistan desert, Pakistan	*Ziziphus mauritiana*	29.63886	72.50068	18	1. Saline (*pH* 8.6)	Mean summer temperature (34°C–38°C)	100–250
	*Ziziphus spina-christi*	29.63336	72.49797	22	2. Saline-sodic (*pH* 10)	Mean winter temperature (15°C–20°C)	
	*Ziziphus nummularia*	29.6356	72.50489	12		Highest temperature (51.6°C)	

Lat. = latitude, Long. = longitude, Dbh = diameter at breast height, Temp. = temperature, Precip. = precipitation.

**Table 2:** Anatomical characteristics of vessels in *Ziziphus* spp.

Species	Vessel diameter (*µ*m)^ns^	Vessel length (*µ*m)	Vessel frequency (no./mm^2^)
*Ziziphus mauritiana*	139.23 ± 3.33^ns^	269.61 ± 1.55^a^	6.01 ± 0.29^b^
*Ziziphus spina-christi*	159.58 ± 12.71^ns^	290.99 ± 2.44^a^	3.45 ± 0.58^b^
*Ziziphus nummularia*	144.29 ± 19.45^ns^	282.3 ± 2.91^a^	9.53 ± 1.03^b^

In the same group, values with the same letter are highly significant at *P* ≤ 0.01; ns = not significant.

**Table 3:** Anatomical characteristics of fibers of *Ziziphus* spp.

Species	Fiber width (*µ*m)	Fiber length (*µ*m)	Wall thickness (*µ*m)	Lumen diameter (*µ*m)
*Z. mauritiana*	14.52 ± 0.06^ns^	930.8 ± 62.58^ns^	4.22 ± 0.81^ns^	10.33 ± 1.11^ns^
*Z. spina-christi*	19.68 ± 1.16^C^	1171.53 ± 90.17^C^	4.33 ± 0.55^ns^	10.16 ± 0.63^ns^
*Z. nummularia*	14.01 ± 0.84^ns^	958.36 ± 34.23^ns^	4.25 ± 0.58^ns^	9.53 ± 0.21^ns^

*C*= Significantly different from the other two values in the same group at *P* ≤ 0.01; ns = not significant.

**Table 4:** Anatomical characteristics of rays in *Ziziphus* spp.

Species	Ray diameter (*µ*m)	Ray height (*µ*m)	Number of cells along ray width	No. of rays per mm^2^	Number of cells along ray height
*Z. mauritiana*	27.44 ± 3.48^ns^	283.01 ± 13.19^ns^	1 ± 0^ns^	17.86 ± 0.33^ns^	6.66 ± 1.64^ns^
*Z. spina-christi*	12.47 ± 2.38^C^	250.82 ± 27.47^ns^	1.26 ± 0.28^ns^	20.60 ± 1.26^ns^	7.53 ± 0.66^ns^
*Z. nummularia*	24.88 ± 2.07^ns^	238.83 ± 29.92^ns^	1 ± 0^ns^	34.33 ± 0.23^C^	9.06 ± 0.89^ns^

*C*= Significantly different from the other two values in the same group at *P* ≤ 0.01; ns = not significant.

**Table 5:** Correlations (Pearson) among the targeted anatomical parameters.

		VL	Vd	VF	FL	FW	FWT	FLD	RH	RD	RF	NOCRH	NOCRW
VL	Pearson correlation	1	0.588 ^∗^	-0.317	0.744 ^∗^	0.768^∗∗^	0.212	-0.312	-0.453	-0.821 ^∗∗^	0.230	0.215	0.630 ^∗^
	Sig. (1-tailed)		0.048	0.203	0.011	0.008	0.292	0.207	0.111	0.003	0.275	0.289	0.035
	*N*	9	9	9	9	9	9	9	9	9	9	9	9

Vd	Pearson correlation	0.588 ^∗^	1	-0.374	0.294	0.601 ^∗^	0.056	-0.069	0.303	-0.414	-0.122	−0.337	0.213
	Sig. (1-tailed)	0.048		0.161	0.221	0.043	0.443	0.430	0.214	0.134	0.378	0.187	0.291
	*N*	9	9	9	9	9	9	9	9	9	9	9	9

VF	Pearson correlation	-0.317	-0.374	1	-0.625 ^∗^	-0.762 ^∗∗^	-0.048	-0.106	-0.284	0.657 ^∗^	0.765 ^∗∗^	0.215	−0.550
	Sig. (1-tailed)	0.203	0.161		0.036	0.009	0.451	0.393	0.230	0.027	0.008	0.289	0.063
	*N*	9	9	9	9	9	9	9	9	9	9	9	9

FL	Pearson correlation	0.744 ^∗^	0.294	-0.625 ^∗^	1	0.897 ^∗∗^	0.186	-0.176	-0.460	-0.967 ^∗∗^	-0.215	0.242	0.911 ^∗∗^
	Sig. (1-tailed)	0.011	0.221	0.036		0.001	0.316	0.325	0.106	0.000	0.289	0.265	0.000
	*N*	9	9	9	9	9	9	9	9	9	9	9	9

FW	Pearson correlation	0.768 ^∗∗^	0.601 ^∗^	-0.762 ^∗∗^	0.897 ^∗∗^	1	0.090	0.014	-0.148	-0.909 ^∗∗^	-0.375	−0.138	0.850 ^∗∗^
	Sig. (1-tailed)	0.008	0.043	0.009	0.001		0.409	0.485	0.352	0.000	0.160	0.361	0.002
	*N*	9	9	9	9	9	9	9	9	9	9	9	9

FWT	Pearson correlation	0.212	0.056	-0.048	0.186	0.090	1	0.117	-0.091	-0.099	0.110	0.490	0.326
	Sig. (1-tailed)	0.292	0.443	0.451	0.316	0.409		0.382	0.408	0.400	0.389	0.090	0.196
	*N*	9	9	9	9	9	9	9	9	9	9	9	9

FLD	Pearson correlation	-0.312	-0.069	-0.106	-0.176	0.014	0.117	1	0.199	0.326	-0.375	-0.641 ^∗^	0.194
	Sig. (1-tailed)	0.207	0.430	0.393	0.325	0.485	0.382		0.304	0.196	0.160	0.031	0.309
	*N*	9	9	9	9	9	9	9	9	9	9	9	9

RH	Pearson correlation	-0.453	0.303	-0.284	-0.460	-0.148	-0.091	0.199	1	0.388	-0.487	-0.554	-0.431
	Sig. (1-tailed)	0.111	0.214	0.230	0.106	0.352	0.408	0.304		0.151	0.092	0.061	0.123
	*N*	9	9	9	9	9	9	9	9	9	9	9	9

RD	Pearson correlation	-0.821 ^∗∗^	-0.414	0.657 ^∗^	-0.967 ^∗∗^	-0.909 ^∗∗^	-0.099	0.326	0.388	1	0.200	-0.235	-0.801 ^∗∗^
	Sig. (1-tailed)	0.003	0.134	0.027	0.000	0.000	0.400	0.196	0.151		0.303	0.271	0.005
	*N*	9	9	9	9	9	9	9	9	9	9	9	9

RF	Pearson correlation	0.230	-0.122	0.765 ^∗∗^	-0.215	−0.375	0.110	-0.375	-0.487	0.200	1	0.445	-0.250
	Sig. (1-tailed)	0.275	0.378	0.008	0.289	0.160	0.389	0.160	0.092	0.303		0.115	0.258
	*N*	9	9	9	9	9	9	9	9	9	9	9	9

NOCRH	Pearson correlation	0.215	-0.337	0.215	0.242	-0.138	0.490	-0.641 ^∗^	−0.554	-0.235	0.445	1	0.067
	Sig. (1-tailed)	0.289	0.187	0.289	0.265	0.361	0.090	0.031	0.061	0.271	0.115		0.432
	*N*	9	9	9	9	9	9	9	9	9	9	9	9

NOCRW	Pearson correlation	0.630 ^∗^	0.213	-0.550	0.911 ^∗∗^	0.850 ^∗∗^	0.326	0.194	-0.431	-0.801 ^∗∗^	-0.250	0.067	1
	Sig. (1-tailed)	0.035	0.291	0.063	0.000	0.002	0.196	0.309	0.123	0.005	0.258	0.432	
	*N*	9	9	9	9	9	9	9	9	9	9	9	9

^∗∗^Significant = 0.01 level (1-tailed). ^∗^Significant = 0.05 level (1-tailed).

**Table 6:** Mesomorphy and vulnerability indices among *Ziziphus* spp.

Species	Mesomorphy index	Vulnerability index
*Ziziphus mauritiana*	6260.81 ± 455	23.21 ± 1.55
*Ziziphus spina-christi*	13825 ± 3328	47.53 ± 11.66
*Ziziphus nummularia*	4285 ± 543	15.16 ± 1.76

**Table 7:** Comparative analysis of arithmetic ratios of targeted *Ziziphus* spp.

Species	Runkel ratio	Flexibility ratio	Slenderness ratio	Solid factor	Rigidity coefficient ratio
*Ziziphus mauritiana*	0.8 ± 0.1	72.66 ± 6.65	66.18 ± 6.08	96871 ± 28653	29.66 ± 7.37
*Ziziphus spina-christi*	0.87 ± 0.1	50.33 ± 1.15	59.88 ± 2.22	335769 ± 74992	21.33 ± 2.08
*Ziziphus nummularia*	0.87 ± 0.1	69 ± 3	68.75 ± 5.02	98383 ± 19101	30 ± 3

## Data Availability

The data will be available on demand.
